# Hereditary Hemorrhagic Telangiectasia: A Rare Cause of Anemia

**DOI:** 10.7759/cureus.5349

**Published:** 2019-08-08

**Authors:** Waseem Jan, Asim Tameez Ud Din, Farooq Mohyud Din Chaudhary, Ahsan Tameez-ud-din, Faisal Nawaz

**Affiliations:** 1 Gastroenterlogy, Nishtar Medical University & Hospital, Multan, PAK; 2 Internal Medicine, Rawalpindi Medical University, Rawalpindi, PAK; 3 Gastroenterology, Nishtar Medical University & Hospital, Multan, PAK; 4 Gastroenterology, Good Hope Hospital, University Hospitals Birmingham, Birmingham, GBR

**Keywords:** hereditary hemorrhagic telangiectasia, telangiectasia, anemia

## Abstract

Hereditary hemorrhagic telangiectasias (HHT), also known as Osler-Weber-Rendu syndrome, is an uncommon genetic disorder. It is inherited as an autosomal dominant disorder with varying penetrance and expression. The diagnosis of HHT requires the presence of at least three out of four clinical criteria. These so-called Curaçao criteria include epistaxis, telangiectasias, visceral involvement, and a family history of HHT in a first-degree relative. Visceral involvement can involve the gastrointestinal (GI) tract, resulting in the development of GI telangiectasias. One of the complications is anemia due to the chronic blood loss from these vascular malformations. Here, we present a case of a 26-year-old male who was diagnosed with HHT. He initially had episodes of epistaxis but now presented to us with features of anemia. According to the patient, he didn’t have epistaxis for the past many months and on his esophagogastroduodenoscopy (EGD) and colonoscopy, there was evidence of multiple small telangiectasias seen in his stomach, duodenum, and colon. He was managed with blood transfusion and was discharged on oral iron supplementation. This is a rare cause of anemia and should be evaluated if other features of HHT are present.

## Introduction

Hereditary hemorrhagic telangiectasia (HHT) is one of the rare genetic disorders. The average prevalence estimated in the United States is 0.3 per 10,000 people. A diagnostic criterion designed for its recognition requires three of the following points to be met: epistaxis, many telangiectasias, the involvement of viscera, and the presence of HHT in a first-degree relative [1]. The symptoms predominantly depend on the age of the patient. Epistaxis is one of the common manifestations in young patients below 25 years of age. In contrast, gastrointestinal (GI) bleed is more common in older-aged patients. The documented prevalence of GI bleed in HHT patients ranges from 13%-25% in various studies [2]. Although the distribution of telangiectasias is variable, the common sites include the face, mouth, and limbs. GI telangiectasias can also develop due to visceral involvement, which can be one of the causes of anemia in these patients [3]. The management of HHT is mainly conservative [4]. Here, we present a young patient who presented to us with anemia due to underlying GI telangiectasias.

## Case presentation

A 26-year-old male patient presented to the Gastroenterology & Hepatology department, Nishtar Hospital, Multan, Pakistan, in July 2019, with the complaint of easy fatigability for the past few weeks.

**Figure 1:**
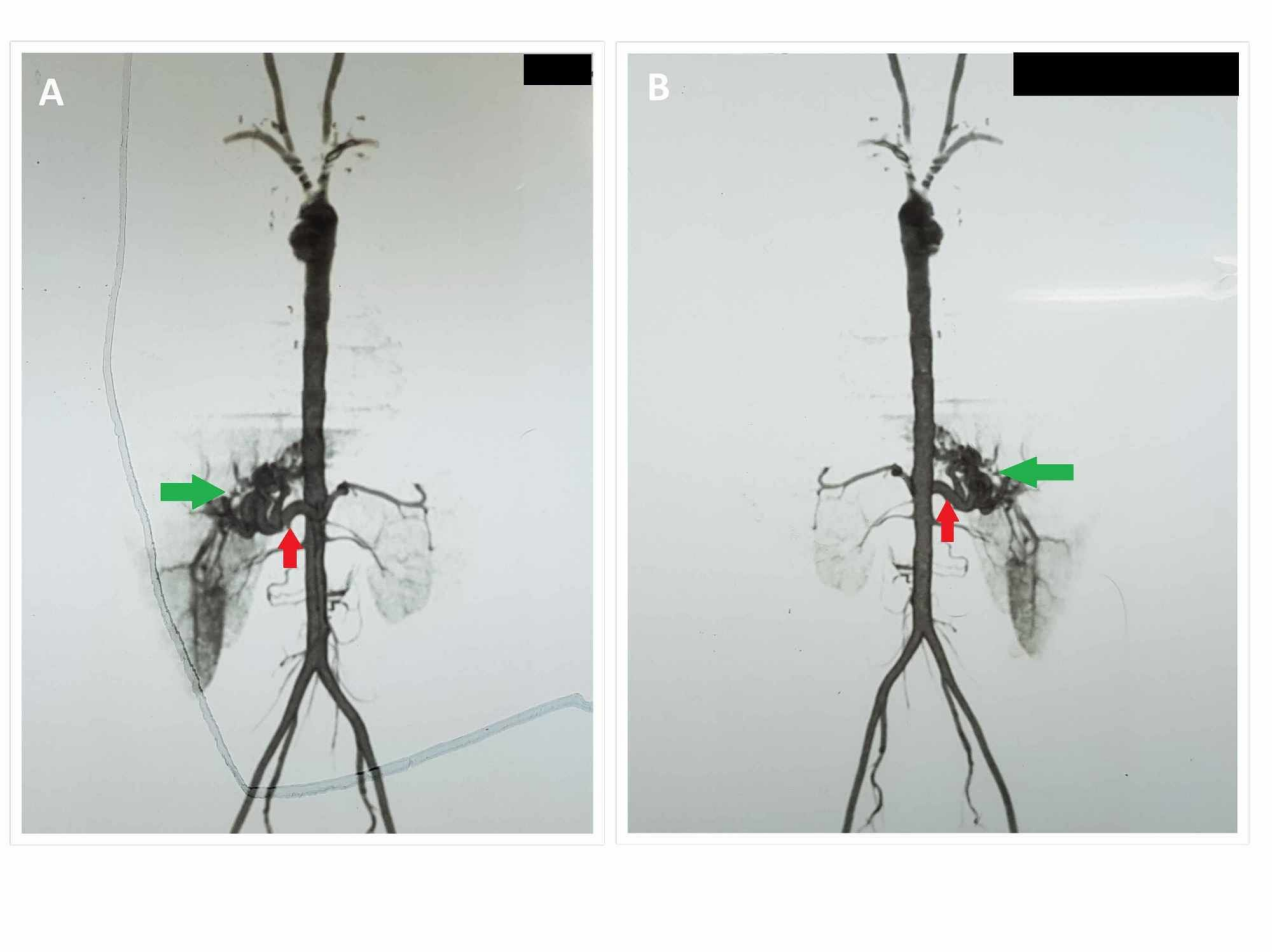
CT angiogram of abdominal vessels taken in supine (A) and prone (B) positions showing significantly dilated and tortuous hepatic artery (red arrow in A and B) with an abnormal cluster of contrast-filled vessels (green arrow in A and B), likely supplied by the hepatic artery and anatomically located in the region of porta hepatis. Early opacification of the portal vein identified. These findings likely represent a hepatic arteriovenous malformation with possible fistulous communication between the portal vein and the hepatic artery. Aorta (thoracic and abdominal) is well-outlined with contrast with normal opacification of its major branches. CT: computed tomography

The patient had a history of recurrent nose bleeds from the last many years. These episodes were more frequent during the winter season. They had no relation to any particular food, activity, or posture. For many years, the patient took herbal medicines from different Hakeems (those who practice alternate forms of medicine). However, his symptoms always recurred. It was not until last year when he had several episodes of severe epistaxis (nose bleeds) that required multiple blood transfusions that he was properly evaluated by a physician. His evaluation led to the final diagnosis of hereditary hemorrhagic telangiectasia (HHT). His esophagogastroduodenoscopy (EGD) in 2018 revealed pan-gastritis. Biopsy of the gastric mucosa showed mild chronic active Helicobacter (H.) pylori-associated gastritis. Colonoscopy at that time was normal. Echocardiography showed normal left ventricular (LV) size with normal left ventricular (LV) systolic function and mild pulmonary artery hypertension. A computed tomography (CT) angiogram of his abdomen (Figure 1) revealed hepatic arteriovenous malformation.

The patient remained well for many months after that and was not taking any regular treatment or drugs. He did not have any episode of epistaxis for many months.

Now, the patient presented to the Gastroenterology & Hepatology department, Nishtar Hospital, Multan, with complaints of easy fatigability for the past few weeks. He was not able to carry out his daily activities. He felt short of breath while walking to the market. He had also noticed a few episodes of black tarry stools during this time. However, there was no history of hematemesis. The patient denied any intake of painkillers, alcohol, illicit drugs, or any type of alternate forms of medicine. There was no history of headaches, altered level of consciousness, fever, cough, blood in sputum, haematuria, diarrhea, or abdominal distension. His family history revealed that his mother had died when he was a boy. She had a history of multiple blood transfusions. However, he was not sure of her diagnosis or cause of death. His siblings were all in good health.

**Figure 2:**
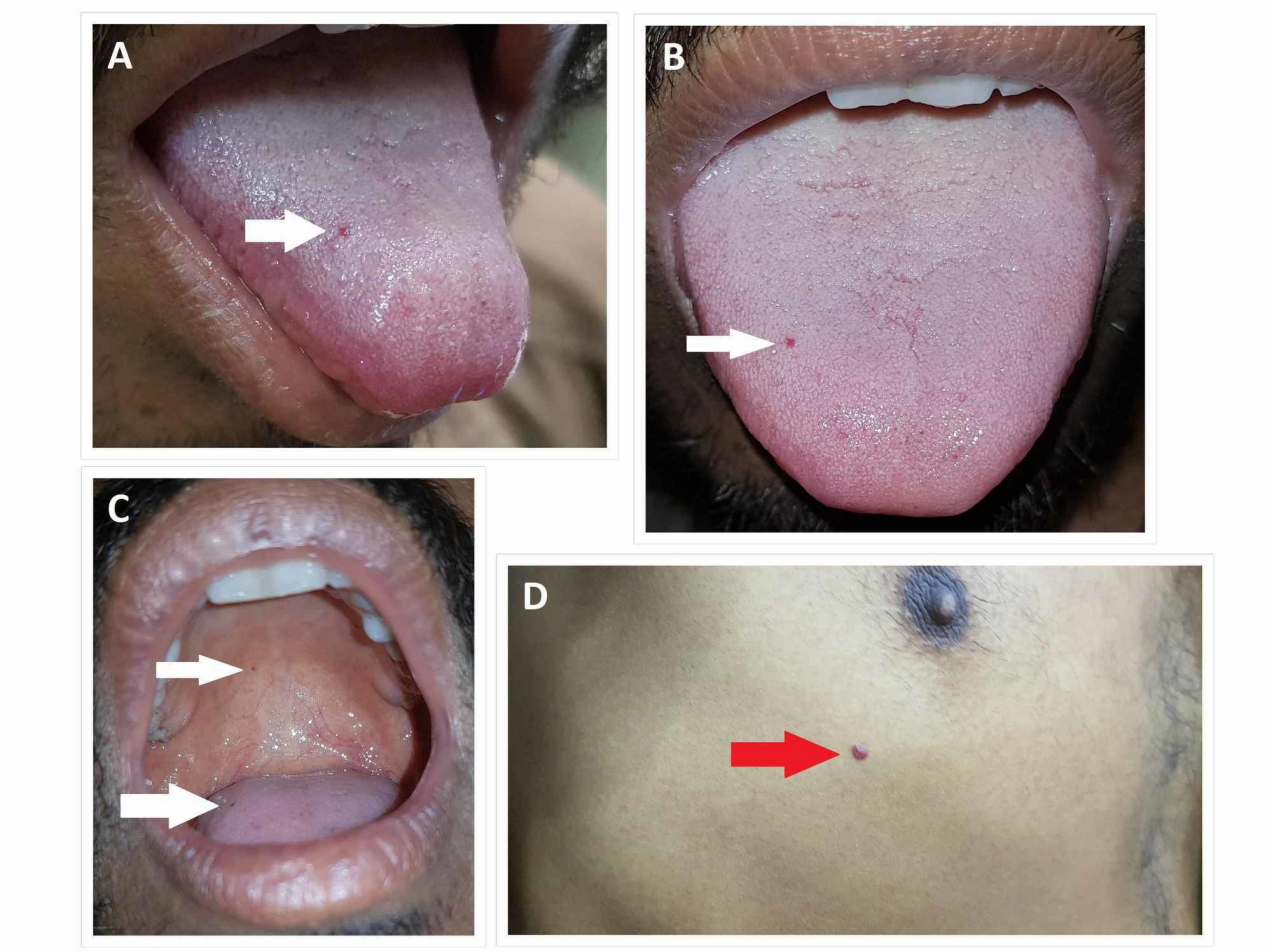
Telangiectasias (white arrows) are seen on the tongue (A, B, C) and palate (C) of the patient. A cherry angioma (red arrow) is seen below the right nipple of the patient (D).

On examination, the patient was pale and there were two small 1 to 2 mm, discrete, red, macular and papular telangiectasias (Figure 2) on his tongue and palate (oral cavity). A cherry angioma (Figure 2) was also found below the right nipple.

His abdominal exam revealed an enlarged liver. His initial workup revealed that he had severe anemia, most likely due to the chronic blood loss through the gut. His hemoglobin was 4.8 g/dl. His stool for occult blood was positive. His other laboratory investigations are shown in Table 1.

**Table 1:** Laboratory investigations WBC: white blood cells; RBC: red blood cells; PCV: packed cell volume; MCV: mean corpuscular volume; MCHC: mean corpuscular hemoglobin concentration; ALT: alanine aminotransferase; AST: aspartate aminotransferase; ALP; alkaline phosphatase; HBsAg: surface antigen of hepatitis B virus; Anti-HCV: antibody to hepatitis C virus; HBeAg: e antigen of hepatitis B virus; Anti-HIV: antibodies to human immunodeficiency virus; Anti-HDV: antibodies to delta virus; HBV DNA: hepatitis B virus DNA levels; INR: international normalized ratio; +ve: Positive; -ve: Negative

Peripheral Blood	Results	Blood Chemistry	Results
WBC	2000/ uL	ALT	11.0 U/L
RBC count	2.19 x 10^6^/uL	AST	18.5 U/L
Hemoglobin	4.8 g/dl	ALP	53U/L
PCV	14.46 %	Bilirubin	1.47 mg/dl
MCV	66 fL	Urea	18.9 mg/dl
MCHC	21.7 g/dl	Creatinine	0.94 mg/dl
Neutrophils	68%	Sodium	135 mmol/L
Lymphocytes	25.3%	Potassium	3.97 mmol/L
Mixed	6.7%	Chloride	103.9 mmol/L
Platelets	165000/uL		
Coagulation tests		Serological Tests	
Prothrombin time	12	HBsAg	+ve
Control	12	Anti-HCV	-ve
INR	1.0	Anti-HIV	-ve
		HBV DNA	500 IU/ml
		HBe Ag	-ve
		Anti-HDV	-ve

**Figure 3:**
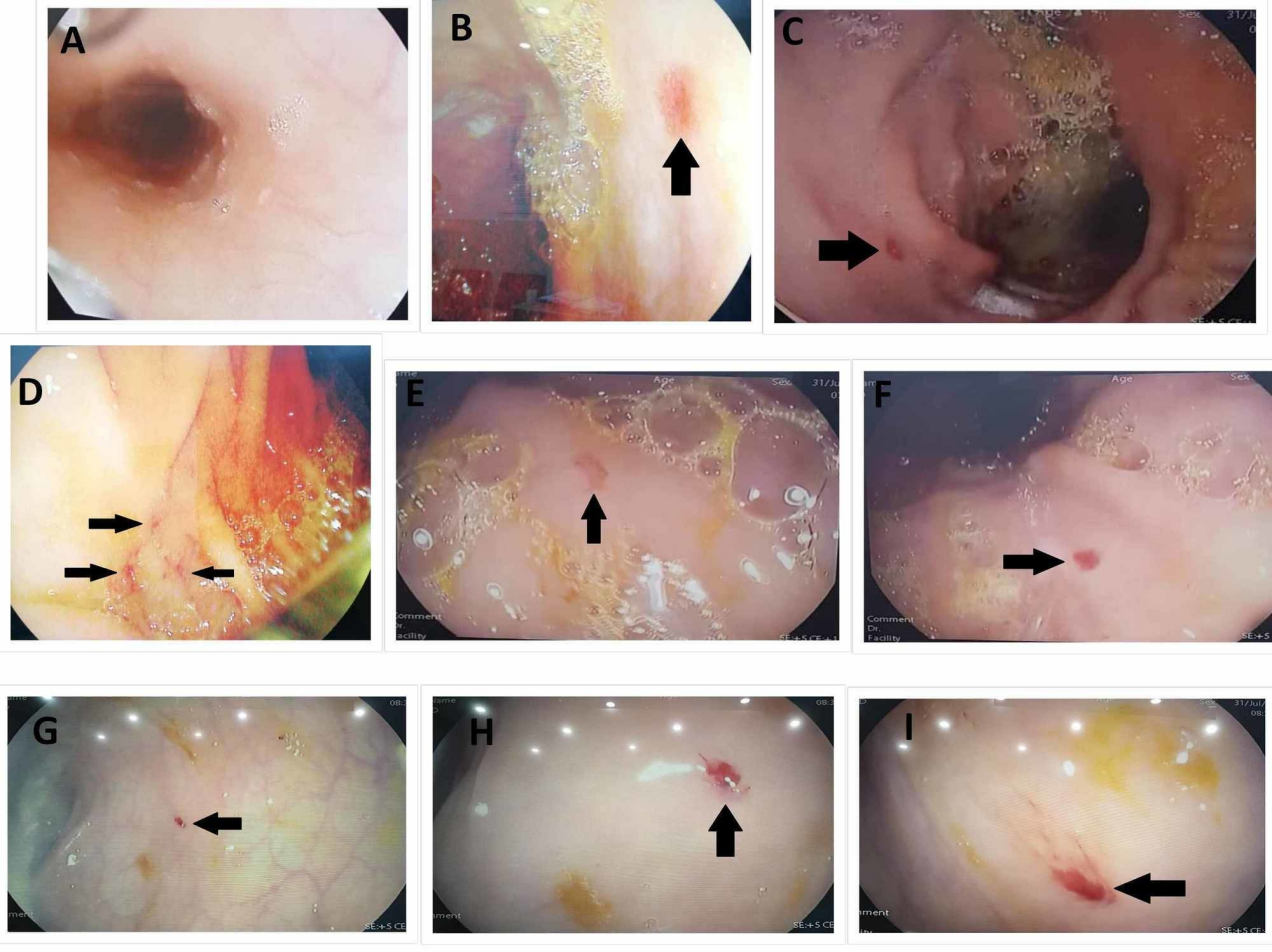
Endoscopic images showing multiple telangiectasias (black arrows) characteristic of HHT in the stomach body (B, C), duodenum (D), fundus (E, F), and the rectum and sigmoid part of the colon (G, H, I). Esophagus (A) is normal. HHT: hereditary hemorrhagic telangiectasia

Ultrasound abdomen showed an enlarged non-echogenic liver. His EGD and colonoscopy revealed multiple small telangiectasias seen in his stomach, duodenum, and colon (Figure 3). His final diagnosis was made as HHT with severe anemia due to GI telangiectasias and chronic hepatitis B infection. After a few days of conservative therapy, which included transfusion of packed red blood cells (RBCs) and iron, the patient was discharged on iron supplements and referred to a specialized center for endoscopic thermal ablation.

## Discussion

HHT is an autosomal dominant genetic disorder. The common clinical features include nose bleed, multiple telangiectasias, and the formation of arteriovenous malformations (AVMs) in different visceral organs, such as the lungs, liver, and brain. Telangiectasias, defined as dilated small blood vessels, can be present in different locations like the lips, mouth, nose, and even in viscera like the gastrointestinal tract. The underlying mechanism of these lesions includes abnormal growth of endothelial cells and vessels. The key pathophysiology is linked to genetic mutations leading to a decrease in transforming growth factor (TGF-ß). Our patient also demonstrated AVMs in liver and telangiectasias in the oral cavity, which consolidated our diagnosis [5].

Anemia in an HHT patient could be present due to bleeding from existing vascular malformations. In some patients, due to excessive nose bleed, there could be the ingestion of a small amount of blood. This can mimic an upper GI bleed with manifestations of Malena and hematemesis [2]. Our patient didn’t have epistaxis for many months, and his EGD and colonoscopy revealed multiple small telangiectasias in the upper as well as the lower GI tract. These findings confirmed the GI telangiectasias as the bleeding source in our patient.

There are multiple articles highlighting this rare disease, including pediatric and adult patients [6-7]. Gastrointestinal bleed is mostly found in patients who are in their fifth decade of life [8]. In contrast to this, our case demonstrated this entity in a young patient.

The treatment of HHT is mainly conservative. Unfortunately, there is no permanent cure for bleeding and anemia in these patients. The therapy revolves around the prevention and acute management of these manifestations, including blood transfusions and iron supplementation. New therapies like hormonal, thalidomide, and bevacizumab have shown promising results but further evaluation is required for their long-term use [5]. Our patient was also managed on these lines and was referred to a specialized center for endoscopic thermal ablation.

## Conclusions

HHT is a rare genetic disorder presenting with epistaxis, multiple telangiectasias, AVMs, and visceral involvement. Gastrointestinal telangiectasias can lead to chronic blood loss resulting in anemia. We present a diagnosed case of HHT who developed anemia due to bleeding from GI telangiectasias. The management is supportive, involving blood transfusions and iron supplementation. This is a rare cause of anemia and should be considered a differential if other features of HHT are present.
